# Green space, social inequalities and neonatal mortality in France

**DOI:** 10.1186/1471-2393-13-191

**Published:** 2013-10-20

**Authors:** Wahida Kihal-Talantikite, Cindy M Padilla, Benoît Lalloué, Marcello Gelormini, Denis Zmirou-Navier, Severine Deguen

**Affiliations:** 1 EHESP School of Public Health–Rennes, Sorbonne-Paris Cité, France; 2 INSERM U1085-IRSET – Research Institute of Environmental and Occupational Health, Rennes, France; 3 Lorraine University Medical School–Vandoeuvre-les-Nancy-France, Vandoeuvre-Les-Nancy, France

**Keywords:** Greenness level, Neighborhood deprivation, Infant mortality, Spatial analysis

## Abstract

**Background:**

Few studies have considered using environmental amenities to explain social health inequalities.

Nevertheless, Green spaces that promote good health may have an effect on socioeconomic health inequalities. In developed countries, there is considerable evidence that green spaces have a beneficial effect on the health of urban populations and recent studies suggest they can have a positive effect on pregnancy outcomes. To investigate the relationship between green spaces and the spatial distribution of infant mortality taking account neighborhood deprivation levels.

**Methods:**

The study took place in Lyon metropolitan area, France. All infant deaths that occurred between 2000 and 2009 were geocoded at census block level. Each census block was assigned greenness and socioeconomic deprivation levels. The spatial–scan statistic was used to identify high risk cluster of infant mortality according to these neighborhood characteristics.

**Results:**

The spatial distribution of infant mortality was not random with a high risk cluster in the south east of the Lyon metropolitan area (p<0.003). This cluster disappeared (p=0.12) after adjustment for greenness level and socioeconomic deprivation, suggesting that these factors explain part of the spatial distribution of infant mortality. These results are discussed using a conceptual framework with 3 hypothetical pathways by which green spaces may have a beneficial effect on adverse pregnancy outcomes: *(i)* a psychological pathway, *(ii)* a physiological disruption process and *(iii)* an environmental pathway.

**Conclusions:**

These results add some evidence to the hypothesis that there is a relationship between access to green spaces and pregnancy outcomes but further research is required to confirm this.

## Background

In developed countries, the leading causes of neonatal morbidity and mortality are adverse pregnancy outcomes such as preterm birth [1], congenital malformations [2], low birth weight [1] and intrauterine growth retardation [3]. Socio-epidemiological research documented a social gradient of infant mortality and stillbirth [4,5]. Infant mortality and its risk factors are more common among women of low socioeconomic status [6,7]. A wide literature describes various deprivation measures related to adverse birth outcome, including composite indices [6,8] and proxy variables of socioeconomic characteristics, such as income [9], level of education [10,11], unemployment [10], occupation [10], percentage of persons below the poverty level [10], homeownership [12] and percentage of immigrants [9]. However, only a few studies have combined individual and the neighborhood socioeconomic status [7,13,14].

Environmental factors have recently been proposed as determinants which could partially explain social health inequalities. Most of these studies focused on environmental nuisances, such as ambient air pollution related to traffic or industry [15-17] and noise [18,19]. Only a few have considered environmental benefits [20]. However, several recent papers have shown that access to green spaces may have a beneficial effect on health [21,22] and may relate to urban socioeconomic inequalities [20].

Two recent reviews have reported that green space, defined as “*open, undeveloped land with natural vegetation, parks or forest*”, have beneficial health effect on morbidity [23] and mortality [20]. The literature suggests various ways in which green space may promote health by encouraging physical activity [24,25] and walking [26], reducing pollution (air pollution [27,28] and noise [29]) and increasing social contact [30]. In addition, a wide literature explored the psychological benefits of green space. By relieving stress [31], green spaces have a positive influence on people’s self-perceived health [32], emotional and mental health [33] and well-being [26]. Some studies have shown that, in addition to promoting psychological health, physical or visual contact with green space can have a physiologically restorative effect. Many health benefits have been reported, such as a reduced incidence of cardio-vascular diseases [34], overweight and obesity [35] and even mortality [20].

To our knowledge, only two teams, one in Spain and one in Portland investigated, the effects of green spaces on adverse pregnancy outcomes [36-39]. An association was reported between living near a green space and birth weight or gestational age. These recent findings highlight the need for research into the relationship between green space and pregnancy outcomes in order to improve our understanding of the underlying mechanisms.

Different socioeconomic groups still have unequal access to green spaces. The Spanish study showed a clear association between living close to a green space and birth weight or gestational age, although only for the group with the lowest level of education [36]. Other authors also reported that people with greater access to green space were less likely to be deprived than those with limited access [40,41]. Green spaces that promote good health may therefore have an effect on socioeconomic health inequalities.

In this context, our study explored the relationship between living close to green spaces and spatial distribution of infant mortality in Lyon metropolitan area, France, between January 2000 and December 2009, and assessed the effect of socioeconomic level on this relationship. We conducted a spatial–scan statistic analysis, which is used for an increasing number of spatial epidemiology applications [42]. The results of this analysis were then discussed using a theoretical model elaborated to explain the possible mechanisms by which green space and socioeconomic level may be related to adverse pregnancy outcomes.

## Methods

### Study setting

The study was carried out in Lyon metropolitan area, an urban area covering 515.96 km^2^ with a population of 1,340,155 in 2009 located in east-central France. The prevalence of infant death over the study period (2000–2009) was on average of 3.5 per 1000 live birth (Max: 4.1‰ birth in 2001, Min: 3.1‰ birth in 2004) [43].

### Health data

The dependant variable is infant mortality, defined as all cases of deaths of infants less than 1 year old. The data was collected from all city halls of each municipality by those involved in the “Equit’Area” project (http://www.equitarea.org). Each case was geocoded on the basis of the parents’ postal address using CAZU software produced by INSEE (National Institute for Statistics and Economic Studies) which assigns street names and numbers to census blocks (2000 inhabitants on average). The exhaustiveness of the death data is 96,5%, by comparing the total number of cases collected from the death and birth registries of the study area City Halls with the cases obtained from the National Epidemiological Center for Medical Causes of Death (CepiDc-Inserm). Due to the mortality statistical system in France, whose smallest spatial resolution scale is the city, no data exists at a census block level, a limitation that forced us to visit the death registries in all municipalities to retrieve the information. It was possible to check that the cases were evenly distributed across the deprivation and greenness scales. Overall, 715 cases of infant deaths in Lyon metropolitan area were collected between January 2000 and December 2009. The CNIL (French National Commission for Digitalized Information and Liberty) gave its permission to retrieve geocode and analyze the health data.

### Neighborhood characteristics

#### Socioeconomic index

Socioeconomic and demographic data (income, level of education, employment, immigration, etc.) were obtained from the 2006 census conducted by INSEE at census block level.

In order to characterize the neighbourhood deprivation level, we used a deprivation index. This measure combines material and social aspects of deprivation to measure the overall socioeconomic status. It includes variables related to education, income, occupation, unemployment, and immigration (see Table 1) to cover and capture the different dimensions of the deprivation.

**Table 1:** Description of the deprivation categories

**Data**	**Characteristics**	**Description**
**Classes of deprivation**	Group 1: low deprivation	Census block with high median income, low proportion of households without a car, low proportion with non-owner-occupied primary residences
	Group 2: moderate deprivation	Census block with median income average, medium proportion of households without a car, medium proportion with non-owner-occupied primary residences
	Group 3: high deprivation	Census block with low median income, high proportion of households without a car, high proportion with non-owner-occupied primary residences

Successive principal-component analyses were used to create the deprivation index based on Lalloue et al. [44]. The measure of neighborhood deprivation was categorized into three groups according to the tertiles of the index distribution (Table 1): low, moderate and high deprivation (Table 1).

#### Green space index

Spatial land cover datasets for Lyon Metropolitan area were sought and processed using ArcMap GIS software (ESRI) to produce a green space index. The definition of green space included natural area (e.g. parks, forest) as these are generally treated as green space in the literature.

Our greenness index represented proportion of the geographical area (km^2^) of green space in the total area of census block.

This index, measured in each census block, was categorized into three groups defined according to the tertiles of the index distribution: low, moderate and high greenness.

### Statistical analysis

#### Spatial methodology

The spatial scan statistics implemented in SaTScan software [45] were used to carry out a cluster analysis to determine the spatial aggregation of infant mortality. This approach showed the presence of high risk clusters of infant mortality named “most likely clusters” and their spatial location. The number of cases in each census block was assumed to follow a Poisson distribution.

The method used by SaTScan imposes a circular scanning window of variable radius (from zero up to 50% of the population size [46]). This circular window was placed at each centroid of the census block and moved across the whole study area to compare the infant mortality rate in the windows with the rate expected under a random distribution. The identification of the most likely clusters was based on a likelihood ratio test [47] with an associated p-value obtained using Monte Carlo replications [48]. The on-line appendix describes the analytical strategy in detail (Additional file 1).

## Results

**Figure 1:**
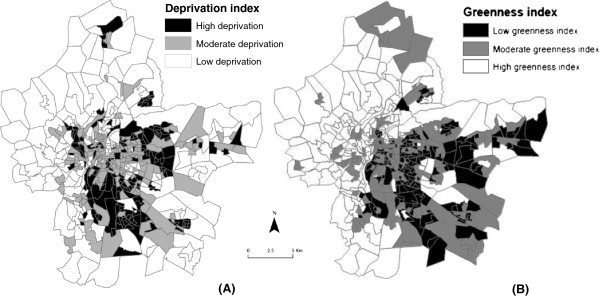
Spatial distribution of the neighborhood socioeconomic index (A); spatial distribution of greenness levels modeled across the Lyon metropolitan area (B).

Figure 1A shows the spatial distribution of the socioeconomic deprivation index. The most wealthy census blocks are located in the center and peripheral parts of the study area, while the most deprived blocks are in the central-eastern and southern areas of the metropolitan area.

Figure 1B shows the spatial distribution of the greenness index, the census blocks with the highest greenness levels being concentrated in the peripheral and western parts of the Lyon metropolitan area and the census blocks with the lowest greenness levels being in the central-eastern and southern parts of the Lyon metropolitan area. The spatial variations of the deprivation and greenness index have similar patterns.

### Spatial analysis

#### Identify high risk clusters of infant mortality

**Figure 2:**
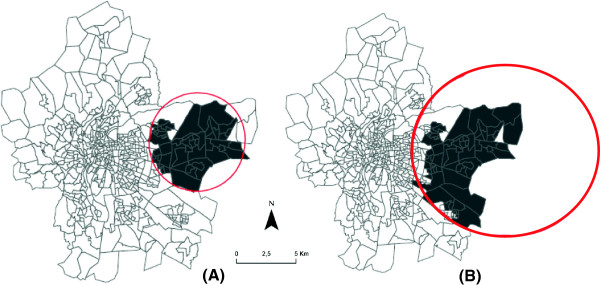
Mapping of the most likely cluster of infant mortality (A), spatial shift of the most likely cluster of infant mortality after adjustment (B).

Figure 2A shows the location of the most likely cluster in the south-east of Lyon metropolitan area, with an infant mortality rate 1.70 times higher than in the rest of the study area (p=0.003). This cluster is composed of 53 census blocks with a population of around 19,401 (Table 2, unadjusted analysis).

**Table 2:** The most likely clusters resulting from the unadjusted analysis (stage 1) and adjusted analysis (stages 2 and 3)

**Most likely cluster**	**Confounders**	**Radius (meter)**	**Census block included**	**Expected cases**	**Observed cases**	**RR**^**a**^	**LLr**^**b**^	**p-value**
**Unadjusted analysis**^**c**^**- Stage 1-**								
	None	5117.81	53	73.30	116	**1.70**	**12.00**	**0.003**
**Adjusted analysis**^**d**^**- Stage 2-**								
	Green space	5117.81	53	76.46	116	**1.52**	**10.06**	**0.01**
	SES level	9258.9	66	85.68	124	**1.54**	**8.74**	**0.06**
**Adjusted analysis**^**e**^**- Stage 3-**								
SES level and greenness level		9258.9	66	85.95	124	**1.50**	**7.60**	**0.12**

^**a**^RR: Relative Risks.

^**b**^LLr: Log Likelihood ratio.

^**c**^Unadjusted analysis, to identify and localize the most likely cluster/s of high risk of mortality, (first step of analysis).

^**d**^Adjusted analysis for greenness level or socio-economic neighbourhood (deprivation index), (second step of analysis).

^**e**^Adjusted analysis for greenness level and deprivation index at the neighbourhood level including the interaction between the two variables, (third step of analysis).

### Adjusted scan statistical analysis

#### Greenness level and spatial distribution of infant mortality

After adjusting for greenness index, the most likely cluster was in the same position (Figure 2A), with a log likelihood ratio reduced from 12 to 10.06 (Table 2, adjusted analysis -stage 2). This indicated that the greenness level only partially explained the excess infant mortality risk found in the south-eastern part of Lyon metropolitan area [46]. However, the cluster was still significant after adjustment (RR=1.52; p = 0.01), indicating that the excess infant mortality risk should be explained by other variables.

#### Neighborhood deprivation level and spatial distribution of infant mortality

Adjusting for deprivation index increased the size of the most likely cluster to 66 census blocks located in the same general location (Figure 2B), with a population of about 21,907 in a radius of 9258.9 m (Table 2, adjusted analysis – stage 2). The risk of infant mortality was 1.54 higher than in the rest of the metropolitan area, the log likelihood ratio reduced from 12 to 8.74 and the cluster of infant mortality became borderline significant (p=0.06), indicating that the socioeconomic index explained a major part of the excess infant mortality shown by the unadjusted analysis [46].

#### Greenness level, neighborhood deprivation level and spatial distribution of infant mortality

After adjustment for greenness and deprivation levels and their interaction, in stage 3 of the analysis (Table 2, adjusted analysis – stage 3), the most likely cluster became not significant (p=0.12) but remained in the same general location (Figure 2A). When interaction between deprivation and greenness levels was included, there was no difference in the results, meaning that both factors had an independent effect.

## Discussion

To our knowledge, this is the first study of the spatial relationship between greenness, deprivation level and infant mortality. Our results revealed that infant mortality rates were not randomly distributed over the study area with a cluster of excess infant mortality in the south-western area of Lyon metropolitan area. After adjusting for greenness and neighborhood deprivation level, high risk cluster of infant mortality disappeared, suggesting that these factors explained the excess infant mortality.

Our findings are consistent with recent studies investigating adverse pregnancy outcomes conducted at the individual level. Two studies highlighted a reduction in the risk of small for gestational age [39] and low birth weight [36-38] associated with a greater surrounding tree canopy [39] or greenness [36-38]. However, no association was observed with gestational age [37-39]. A recent paper reported that living close to green space had various maternal benefits including a decrease in musculoskeletal discomfort, reduced incidence of muscle cramps and lower limb edema [49]. However, the exact mechanisms of these beneficial effects were not established.

The present study provided some additional empirical support for the potential role of access to green space in reducing the risk of adverse pregnancy outcomes.

**Figure 3:**
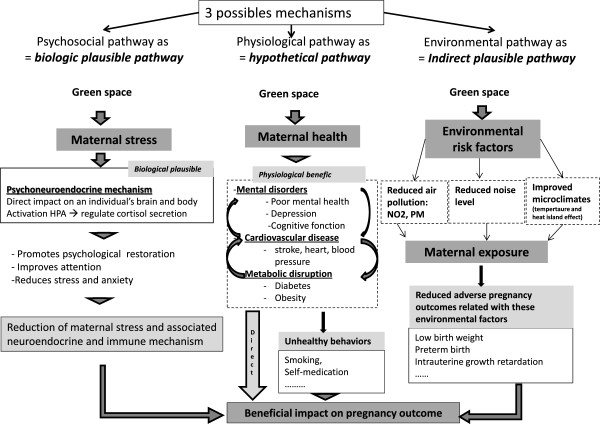
A conceptual model of mediating variables and their hypothesized association with pregnancy outcomes.

A conceptual model of the mediating variables associated with green space and of their hypothetical relationship with pregnancy outcomes is proposed below (Figure 3).

We propose hereafter 3 hypothetical pathways through which green spaces may have a beneficial effect on pregnancy outcomes.

### Psychological pathway as a possible biological pathway

The main mechanism by which green space may be associated with a favorable pregnancy outcome is stress reduction. A large number of experimental studies have produced strong evidence of the positive effect of nature on recovery from stress and attention fatigue [22,31]. Contact with natural environments promotes psychological restoration [50] and reduces stress and anxiety [51-53]. Green space has a positive effect on stressful life events including mood and stress levels not only by providing people with a pleasant view but also by encouraging physical activity [54,55] and social contact [30,56].

There is evidence that perception of green space has a direct impact on a person’s brain and body through psycho-neuroendocrine mechanisms, including the functioning of the hypothalamic pituitary adrenal axis which regulates cortisol secretion and whose deregulation is associated with a range of adverse pregnancy outcomes.

The main theoretical model for these responses, known as Ulrich’s psychoevolutionary model [52,53], has been confirmed by several experimental studies that revealed that being in or viewing green space was linked to a reduction in physiological manifestations of stress, including heart rate, blood pressure, skin conductance and muscle tension [53,57]. Surprisingly, these findings were not reported in other papers [58].

Some beneficial effects of green space may operate through reduction of maternal stress and the neuroendocrine and immune mechanisms which may alter feto-maternal exchanges [59,60], causing limited fetal nutrition and/or oxygenation, leading to a reduction in fetal growth [61,62] and preterm birth [63].

### Physiological disruption as a hypothetical pathway

The second pathway posits that green space may promote maternal health by encouraging physical activity and facilitating social contact. There may be a number of underlying mechanisms. Firstly, by providing the opportunity for physical activity, access to green spaces may improve maternal cardiovascular activity [49,64] which, by a number of biological pathways, may reduce blood pressure, decrease the concentrations of proinflammatory cytokines and leptin in the peripheral circulation, reduce oxidative stress and improve plasma lipids and lipoprotein concentrations [64]. A large number of studies have produced strong evidence of these positive effects including reduction in the risk of hypertensive disorders [25,65] and the risk of preeclampsia [64,66], both conditions that are associated with preterm birth [67], low birth weight [68] and infant mortality [69,70].

Secondly, by encouraging physical activity, green space may have positive effects on metabolic disorders including weight gain [25] and diabetes [71] during pregnancy. The weight gain during pregnancy has significant health implications on the newborn [72]. Maternal obesity and a sedentary lifestyle during pregnancy have been associated with preterm birth [25,73] and increased risk of congenital anomalies, a leading cause of stillbirth and infant mortality, and important contributors to preterm birth and early childhood morbidity [72].

A recent meta-analysis and epidemiological studies documented that women who are physically active during pregnancy have a 24% lower odds of developing gestational diabetes than inactive women [71] and that the risk of spontaneous preterm birth increased with increasing levels of pregnancy [74].

Thirdly, through an association between physical activity [75,76] and social contact [33,77] on the one hand and mental health on the other, including well-being, mood and depression/insomnia during pregnancy, green space may reduce mental disorders and their effects on adverse outcomes [25,78,79].

#### Environmental pathways, as an indirect pathway

The third hypothetical pathway is the effect of green space on the living environment of pregnant women. Recent studies reported that green space had beneficial effects on environmental factors such as *(i)* ambient air pollution, *(ii)* noise levels and *(iii)* temperature which may lead to adverse pregnancy outcomes.

Green space is associated with lower personal exposure to particulate matter (PM_2.5_) [37]. Broad leaved woodland reduces ambient air pollution and tree-lined streets have around a quarter of the particle concentrations of streets without trees [80,81]. Other studies showed that urban trees, particularly low VOCs emitting species, can reduce urban ozone levels [82-84]. In 2000, Nowak [85] described four main processes by which vegetation may affect air quality: (i) temperature reduction and other microclimatic effects, (ii) removal of air pollutants, (iii) emission of volatile organic compounds and tree maintenance emissions, (iv) energy effects on buildings. Vegetation may play a variety of roles as a physical filter for harmful gases and particulate matter [80,81]. By reducing air temperature, radiation and absorption, tree transpiration and tree canopies can improve air quality because the emissions of many pollutants, including ozone-forming chemicals, are temperature dependent. Trees also improve air quality by reducing energy use and pollutant emissions from power plants [85,86]. Such mechanisms which reduce maternal exposure to hazardous air pollutants may be the means by which living close to green space may have positive effects on pregnancy outcomes [87].

Exposure to noise during pregnancy has been associated with a higher risk of preterm birth [88,89] and low birth weight [90,91]. Green space may reduce environmental noise and so promote a better psychosocial maternal environment that reduces the risk of adverse pregnancy outcomes. Although there is little research establishing the actual benefits of urban green space as a distance barrier to environmental noise, recent papers suggest that green space, particularly trees and large shrubs, is able to mitigate noise in urban areas by providing a barrier to screen out noise [92,93]. "Noise buffers" composed of trees and shrubs may reduce noise by up to 15 db [81]. It is also suggested that trees in urban areas may absorb some traffic noise [29]. In addition, perceived intrusion of noise from traffic can be reduced by vegetation obscuring the noise source and associated traffic movement [81]. In 2007, Gidlöf-Gunnarsson and Öhrström proposed a brief conceptual model for the role of green space on noise annoyance, behaviors and perception of the residential soundscape related to road traffic noise [94].

Our hypothesis highlights the complexity of the mechanisms which link green space to pregnancy outcomes and suggests that other factors such as the socioeconomic status of pregnant women [20,31] may interact to promote or reduce the beneficial effects of green space. Several studies describe disparities in the degree of access to green space according to individual or neighborhood socioeconomic status. In general, deprived neighborhoods in urban areas have fewer parks and walking trails and poorer access to green space in comparison with non-deprived areas [40,41,95,96]. Proximity to and usage of green space depend on the level of education or on income [97]. People living in deprived neighborhoods are less likely to make use of green spaces because they do not perceive the need to do so [98,99], although this has been challenged by other authors.

The ecological nature of our study design did not allow us to assess the usage of green space by people in Lyon area population. Another limitation of our study was the construction of the greenness index. The information on topography or land cover used to construct the index did not distinguish between different types of vegetation, which may affect the pathways proposed for the effects of noise or ambient air pollution. Unlike the Normalized Difference Vegetation Index (NDVI) which measures small-scale green spaces in a standardized way, and other more specific synthetic measures of greenness, our index only measured the degree of greenness in each census block.

Finally, because the outcome of interest is rare, the statistical power of this study is limited. Despite these limitations, the results are consistent with those based on the more detailed NDVI index [38].

## Conclusion

These results add evidence to the relationship between access to green space and pregnancy outcomes. Policies that ensure an equitable distribution of green spaces within urban areas may help to promote fair access to healthy environments. Further studies should be carried out on the effect of access to green spaces on pregnancy outcomes to document the mechanisms involved.

## Abbreviations

RR: Relative risks; LLr: Log likelihood ratio; PM 2.5: Particulate matter < 2.5 μm.

## Competing interests

The authors declare they have no competing financial interests.

## Authors’ contributions

WK undertook the spatial analysis, produced the map and conceptual model, drafted the paper and carried out the literature review. CP collected health data, geocoded the cases to the IRIS level, contributed to interpret the results and draft and finalize the paper. BL implemented the statistical model and helped to finalize the paper. MG carried out the literature review and helped to draft and finalize the paper. DZ-N, Head of the Environmental and Occupational Health Department at the EHESP and co-principal investigator of the Equit'Area Project, was responsible for quality assurance and rigor in the data analysis, reviewed the drafts of the article and contributed to finalize it. SD, principal investigator of the Equit'Area project studying the role of environmental exposure on health inequalities, monitored the general work, helped with the analysis and interpretation of the results and contributed to draft and finalize the paper. All authors read and approved the final manuscript.

## Pre-publication history

The pre-publication history for this paper can be accessed here:

http://www.biomedcentral.com/1471-2393/13/191/prepub

## Supplementary Material

Additional file 1 Analytical strategy and results interpretation. Click here for file
