# Supplementary material for: Green space, social inequalities and neonatal mortality in France
Source: BMC Pregnancy Childbirth. 2013 Oct 20;13:191. doi: 10.1186/1471-2393-13-191 (PMC4015785; doi:10.1186/1471-2393-13-191)
Supplement: Additional file 1 — Analytical strategy and results interpretation. [file 1471-2393-13-191-S1.docx]

**Analytical strategy and results interpretation-**

In this approach, the null hypothesis (H_0_) tested is that the risk of infant mortality is homogeneous in the study area; in other words, the expected infant mortality rate would be randomly distributed in space (Kulldorff 2005). The alternative hypothesis (H_1_) is that there is an elevated risk of infant mortality within a cluster (one or several close census blocks) compared with census blocks outside the cluster; it is called “the most likely cluster”. When the test is statistically significant, it means that the infant mortality rate is not randomly distributed in the study area, here the Lyon Metropolitan area, i.e. that the identified cluster of census blocks presents a significant increase of infant mortality compared to the other census blocks located outside the cluster (Kulldorff and Nagarwalla 1995).

The models were adjusted on co-variates, and three major criteria were used to reject or not the H_0_ hypothesis according to the location of the cluster, its statistical significance and the likelihood ratio value of each model:

- When, after adjustment, the most likely cluster remains in the same location and their likelihood ratio decreases, the interpretation is that the variable(s) incorporated in the model explain(s) partially the excess risk (Kulldorff et al. 1997);
- when the most likely cluster shifts (changes in location), this suggests that the covariate(s) in the model explain(s) the excess risk of the cluster (Kulldorff et al. 1997). In addition, another cluster is identified;
- when the most likely cluster disappears totally, it means that the adjusted infant mortality risk is distributed randomly in space.

Thus, spatial analyses were performed in three step by step stages:

1. unadjusted analysis, to identify and localize the most likely cluster/s of high risk of mortality,
2. adjusted analysis for greenness level or socio-economic neighbourhood (deprivation index),
3. adjusted analysis for greenness level and deprivation index at the neighbourhood level (including the interaction between the two variables).

When we introduced both the greenness and socio-economic levels, we also included an interaction term. Because SatScan does not allow to accommodate an interaction term in the model, we created several dummy variables combining the deprivation and the greenness categories.

Kulldorff M. Information Management Services, Inc. SaTScan: software for the spatial, temporal, and space-time scan statistics, version 6.0. 2005. Available http://www.satscan.org/.

Kulldorff M, Feuer EJ, Miller BA, Freedma LS. Breast Cancer Clusters in the Northeast United States: A Geographic Analysis. Am J Epidemiol 1997; 146:161–170.

Kulldorff M, Nagarwalla N. Spatial disease clusters: detection and inference. Stat Med 1995; 14:799–810.
